# Overcoming Transportation Barriers for Low-Income Individuals with Chronic Conditions: Identifying Barriers and Strategies in Access to Healthcare and Food as Medicine (FAM)

**DOI:** 10.3390/healthcare13222869

**Published:** 2025-11-11

**Authors:** Hyesu Im, Fei Li, Shanae Stover, Carlie Abel, Janee Farmer, Carlos M. García, Jenna-Ashley Lee, Christopher K. Wyczalkowski

**Affiliations:** 1 Urban Studies Institute, Georgia State University, Atlanta, GA 30303, USA; feili@gsu.edu (F.L.); cmgarcia@atlantaga.gov (C.M.G.); 2 Sociology Department, Georgia State University, Atlanta, GA 30303, USA; sstover2@student.gsu.edu (S.S.); jennaashleymlee@gmail.com (J.-A.L.); 3 School of Social Work, Georgia State University, Atlanta, GA 30303, USA; carlie.abel@gmail.com (C.A.); janeefarmer25@gmail.com (J.F.); 4 Department of Public Management and Policy, Georgia State University, Atlanta, GA 30303, USA; cwyczalkowski1@gsu.edu

**Keywords:** transportation barriers, healthcare access, food as medicine, chronic disease management, qualitative research

## Abstract

**Background/Objectives:** Transportation is a critical social determinant of health with direct impacts on healthcare access and utilization. This study examines transportation challenges faced by low-income individuals with chronic conditions who participated in the Food as Medicine (FAM) program offered by their primary care provider and explores the strategies they employ to overcome those challenges, particularly during the COVID-19 pandemic. **Methods:** We conducted semi-structured interviews with 36 FAM participants from Grady Health System in Atlanta, Georgia between May 2022 and October 2023. Interviews explored their ability to access routine care, FAM, and healthy food as prescribed by their physicians and nutritionists, as well as how the COVID-19 pandemic affected their transportation challenges and solutions. **Results:** Participants reported various transportation barriers including long wait times, delays, cost burdens, unreliable services, and coordination failures, which contributed to missing doctor appointments and FAM attendance. To overcome those challenges, participants planned trips in advance, used multiple transportation options, relied on social networks, or reduced and sometimes forwent trips. The COVID-19 pandemic limited their accessibility to healthcare, FAM, and healthy food options by reducing business hours and disrupting transportation services. Alternatives such as telemedicine and online ordering were less utilized due to distrust, dissatisfaction, and limited digital literacy. **Conclusions:** Transportation barriers can substantially restrict healthcare and food access for low-income individuals managing chronic conditions, especially during public crises that may lead to service disruptions. Transportation assistance that accommodates individuals’ financial circumstances and health conditions, implemented through collaborative efforts of healthcare institutions, transportation agencies, and governments, is essential to facilitating chronic disease management and reducing health disparities.

## 1. Introduction

Transportation is widely recognized as a fundamental social determinant of health (SDOH), influencing access to healthcare [1,2,3,4,5], food [6,7,8,9], and other essential resources that contribute to overall health and well-being [10,11]. Transportation barriers to healthcare and essential resources often disproportionately affect low-income populations and racial and ethnic minorities [4,6,12,13], driven by factors such as residential segregation [14], uneven investment in infrastructure [15], inadequate transit services, and high transportation costs [2,4]. These barriers can result in late arrivals, cancellations, or missed appointments [4,13,16], as well as delayed or incomplete medication adherence [4], hence limiting the efficacy of medical treatment and efficiency of the healthcare system, often resulting in inadequate care and worsening health outcomes [4,17]. Specifically, missed or delayed appointments are associated with increased hospitalization and emergency department visits [18] and higher healthcare expenditures [19]. Therefore, reliable transportation and physical access to healthcare and related resources are crucial for healthcare utilization and chronic disease management, especially for low-income patients.

This study examines transportation barriers among low-income individuals with two diet-related chronic health conditions, diabetes and hypertension, who participate in a Food as Medicine (FAM) program that offers dietary prescriptions, nutrition education, and biweekly free grocery pickups. For patients with diabetes, hypertension, and many other chronic health conditions, dietary changes and interventions are often as important as medication and other treatments [20,21]. Ensuring food access and security can be an integral part of patient care and disease management planning [22,23]. Limited food access is closely linked to adverse chronic health conditions [24,25] and greater healthcare utilization and costs for food-insecure individuals [22,26]. Moreover, the same transportation barriers that limit healthcare access often limit food access [7,9,27,28]. Therefore, we consider access to FAM and fresh, healthy food along with healthcare access in this study as a critical component of chronic disease prevention and management [20,24,29,30].

Transportation barriers to healthcare and healthy food may include long distances and travel times, lack of transit access or direct transit routes, long wait times, infrequent or unreliable services, high costs, and safety or health concerns. Many of these barriers were exacerbated during the COVID-19 pandemic, which caused widespread disruptions in transportation services and business operations. Lockdowns and public health restrictions in the early stages of the pandemic, along with individuals’ fear of COVID exposure, contributed to sharp decreases in out-of-home trips and activities [31,32], while many traditionally face-to-face businesses and activities, including healthcare services, developed online and remote alternatives. At the same time, public transit services were reduced, further constraining mobility options for transit-dependent populations [33]. These changes were experienced differently across socioeconomic groups. Low-income individuals and those with limited educational attainment were less likely to reduce their transit use due to limited remote work options and continued dependence on public transportation [34]. Among low-income individuals with chronic health conditions, research found that the frequency of medical and grocery trips remained relatively stable during the pandemic, as they had little choice but to continue these activities despite limited transportation alternatives [35]. In terms of transportation barriers, for individuals with complex health needs or disabilities, the pandemic often intensified preexisting transportation barriers [17,36].

Building on prior research, this study examines the transportation barriers that low-income individuals with chronic health conditions face, and the coping strategies they employ to overcome those challenges. We investigate the following research questions:

1. What transportation barriers do low-income individuals with chronic health conditions encounter when accessing healthcare, Food as Medicine, and healthy food for chronic disease management?
2. What coping strategies do they employ to overcome these transportation barriers?
3. How did the COVID-19 pandemic reshape transportation access for low-income patients and their coping strategies?

**Figure 1:**
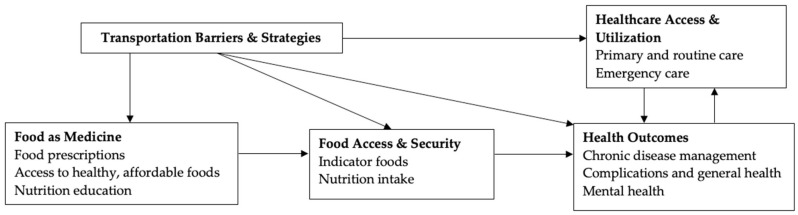
Conceptual Framework.

Figure 1 presents the conceptual framework for this study. It shows how transportation barriers and coping strategies influence healthcare and food access, which in turn affect health outcomes.

We conducted 36 semi-structured interviews with FAM participants at Grady Memorial Hospital in Atlanta, Georgia, between May 2022 and October 2023. Participants described how high costs, long delays, and unreliable transportation services affected their medical and food acquisition trips. They also shared diverse coping strategies in response to these barriers, such as chaining multiple medical appointments in one trip, relying on family for rides, or forgoing outings altogether. These experiences highlight that transportation is a barrier to care and a critical factor that determines the success of chronic disease management and nutrition support. Understanding transportation barriers and coping strategies enables us to identify transportation needs contingent upon individuals’ health conditions and life circumstances, thereby informing the design of tailored, health-promoting interventions.

## 2. Data and Methods

### 2.1. Food as Medicine (FAM) in Atlanta

In August of 2020, Grady Health System (Grady hereafter), Atlanta Community Food Bank, and Open Hand Atlanta together launched Food as Medicine (FAM) in Atlanta, Georgia [37]. FAM is a global initiative that addresses food insecurity and diet-related chronic disease management simultaneously through dietary interventions [38]. The FAM program adopted a multipronged approach, combining food prescriptions with nutrition education and cooking classes targeting patients with stage 2 hypertension and/or uncontrolled diabetes. Patients who qualify for the FAM program would have reported food insecurity and at least one of the qualifying health conditions: (1) blood pressure higher than 140/90 mm Hg, or (2) hemoglobin A1c ≥ 9.0. Eligible patients are referred to the food pharmacy by their primary care or diabetes clinics in the Grady network [20]. Referred patients can then sign up for a three-month episode of FAM participation, which can be renewed for up to 12 months [20]. FAM participants can pick up fresh fruits, vegetables, and other plant-based food prescribed for their conditions every two weeks for free at the food pharmacy and attend nutrition and cooking classes during each three-month episode [20].

Grady is the safety net healthcare provider in metro Atlanta and serves a substantial portion of uninsured and underinsured patients. Its flagship facility, Grady Memorial Hospital, is located in downtown Atlanta and provides care to the low-income communities and residents of Fulton and DeKalb Counties, the two most urban counties with significant impoverished populations in metro Atlanta. The food pharmacy, located at Grady Memorial Hospital, is directly served by a bus stop and has a rail station a few blocks away [39]. By 2025, the FAM program at Grady has served over 3000 patients [40].

### 2.2. Data Collection

The research team partnered with Grady and the FAM program to study how transportation affected healthcare utilization, FAM participation, and chronic disease management for low-income patients during the COVID-19 pandemic. This study is linked to a broader transportation research project that began in August 2020 along with the FAM program and examined the role of transportation in healthcare utilization and health outcomes among FAM participants. Other important partners include the Metropolitan Atlanta Rapid Transit Authority (MARTA), the local public transit agency, and the Atlanta Regional Commission (ARC). For a detailed description of the broader project and its methodology, see [35]. Although the study involved multiple institutional partnerships, the research design and qualitative analysis were independently developed and conducted by our research team without the involvement of institutional partners.

Participants were recruited through purposive sampling from the FAM program, which serves patients with chronic conditions. Two recruitment strategies were used: first, interviewers conducted consecutive telephone outreach to 337 FAM participants from the transportation research project (14 interviews completed); and second, the food pharmacy navigator distributed flyers and compiled contact information from interested patients (71 potential participants, 22 interviews completed). Eligible interviewees included current or former FAM participants who were 18 years of age or older and could speak English fluently. Between May 2022 and October 2023, we conducted in-depth interviews with 36 participants (14 from the transportation research project, 22 from on-site recruitment). Table 1 shows an overview of the participant characteristics.

The semi-structured interviews lasted approximately one hour and focused on participants’ daily travel patterns, particularly for medical and food-related trips. Questions addressed the transportation barriers participants faced, how these barriers affected their access to healthcare and healthy food, and the strategies they used to overcome or circumvent these barriers. Participants also reflected on how the COVID-19 pandemic affected their experiences with healthcare, food, and transportation access. We did not ask participants to distinguish their experiences by pre- and post-pandemic phases; instead, they were encouraged to speak freely about the pandemic’s overall impacts. In addition, they were encouraged to elaborate on related experiences and concerns beyond the interview prompts. Participants reported using diverse modes of transportation, including public transit (MARTA buses and trains), MARTA Mobility (which is a door-to-door, on demand paratransit service for passengers with disabilities), rides by family or friends, senior transportation services provided by local governments (e.g., Fulton County’s “Common Courtesy” program that utilizes Uber and Lyft providers [41]), non-emergency medical transportation provided by insurance, and personal vehicles. Modes of transportation used varied depending on participants’ health status, physical mobility, financial resources, and trip purpose.

### 2.3. Data Analysis

We used GoTranscript [42] to transcribe all interview recordings, and each transcript was reviewed and hand-corrected by the original interviewer for accuracy. We then analyzed the transcripts in NVivo 14.24.3. We performed a thematic analysis to capture common themes within the interview data, beginning with a descriptive coding process in which coders assigned topic-based labels to text segments to capture the primary subject matter. In the initial phase, five research team members independently coded a shared subset of transcripts without relying on a pre-established coding framework. This inductive approach allowed each coder to generate preliminary codes based on their own interpretations of the participants’ narratives. The preliminary coding phase revealed key themes spanning multiple domains, including daily life, emotions, health, life circumstances, social relationships, participation in the study, and systemic issues, such as food, housing, medical care, transportation, and social services, as well as technology use.

**Figure 2:**
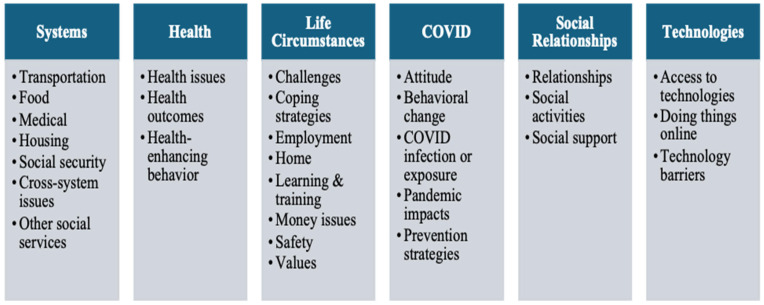
Focused Code Themes and Subthemes.

After completing this open coding phase, the research team collaboratively reviewed and synthesized the individually generated codes to develop a comprehensive codebook. Once finalized, six coders applied this consolidated coding framework uniformly across the complete set of transcripts. Thematic saturation was determined when coders found that no new codes emerged during the coding of later transcripts and all participant experiences could be adequately captured by the existing codebook. The research team confirmed saturation through collective review and discussion. Intercoder reliability was assessed to ensure coding consistency (average percent agreement = 95.9%; average Cohen’s K = 0.66). The average Cohen’s Kappa is relatively low despite high percentages of agreement, likely due to imbalanced data with some codes only assigned to a small number of cases. Figure 2 summarizes the selected themes and subthemes. Quantitative summaries of coding results for the selected key codes, including code frequency, code co-occurrence matrix, and a tree map, are provided in Supplementary Tables S1 and S2 and Supplementary Figure S1. For methodological transparency, we included the consolidated criteria for reporting qualitative research (COREQ) checklist in Supplementary Table S3 [43]. In this analysis, healthcare access encompasses medical visits, pharmacy use, and other medically related travel, including visiting food pharmacy given its location in Grady hospital, while food access includes grocery shopping activities outside the FAM food pharmacy.

## 3. Results

### 3.1. Transportation Barriers

#### 3.1.1. Financial Constraints

Many participants identified transportation costs—for public transit or private vehicle use—as a major barrier to accessing healthcare and food. MARTA fare for buses and trains is $2.50 for a one-way trip; senior citizens (65 or older), disabled, and Medicare recipients are eligible for a reduced fare of $1 [44]. MARTA Mobility fare is $4 for a one-way trip [45], with an application process that consists of a two-part questionnaire to be completed by both the passenger and the passenger’s healthcare provider, an in-person interview, and possible assessment to determine eligibility [46]. Common Courtesy, the contractor that provides Uber/Lyft based senior transportation services for Fulton County, has a $1 per trip fare but with an 8-ride limit each month [41].

Public transportation expenses were a frequent concern among interviewees. One participant remarked, “It’s the bus fare thing that I have a problem with. The bus fare and stuff. I don’t think, I don’t have much bus fare all the time” (P033). Another echoed this burden even under reduced fare programs, stating, “The cost of a MARTA Pass is very expensive. Even if you did a half fare” (P011).

Even among those with access to a private vehicle, financial challenges remained. High gas prices, parking fees, and rising insurance costs often limited their mobility. One participant shared, “*At this time with gas being high, I’ve limited my going out because of the expense of gas, it’s very expensive*” (P009). Another emphasized the difficulty of sustaining car ownership on a fixed income: “*I have a car. It’s hard to keep it on the road because insurance for the vehicle is going up. Then when you’re on a fixed income, it’s kinda rough*” (P019).

#### 3.1.2. Long Wait Times and Delays

When transportation services were available, long wait times and service delays posed significant barriers to timely and reliable healthcare access. Participants frequently described prolonged waits or missed pickups, which led to missed appointments or left them waiting outside for extended periods, often with considerable frustration.

Public transit was especially burdensome due to infrequent schedules and long headways. One participant emphasized the extent of delays: “*That public transportation! It takes so long before you know you got to wait. You got to wait. If I stand out here on Pryor Street, before the bus come back there, it gonna take about three hours*” (P004). Others described challenges with paratransit services. For instance, one participant explained:

*The challenging is like when I call for MARTA Mobility, and then I tell them a time that I need to get picked up from Grady. Then if I don’t get picked up, they’ll put me on will call, and that’s a challenge because a lot of times I have to wait for two hours or an hour, and it’s like I just want to get home.* 

(P010)

In some cases, these delays had direct consequences for their medical appointments. One participant recounted:

*The timing because sometimes you can be on the MARTA or the bus or whatever and accidents happen or things happen or whatever. I was on my way to Emory [hospital] I had an appointment there and the appointment may be scheduled at ten o’clock. They got me there around 10:40. That means Emory when you’re late, that’s a $25 payment.* 

(P035)

As a result, the participant had to reschedule the appointment and incurred a $25 late fee. Technology barriers further compounded these issues. Real-time service updates were often inaccessible to those unfamiliar with smartphone apps or digital platforms: “*They told me I could get a MARTA app. I had stood at Hightower station one time. She said that they changed the times of this bus. I heard you say you were waiting on the three. I said, yes, ma’am. She said, it’s not running for the next four hours. I have been standing there, she said, do your MARTA app. I said, Ma’am, I don’t know how to do that. I don’t know what you’re talking about. What are you saying?*” (P018)

#### 3.1.3. Systemic Issues in Transportation Coordination

Beyond delays, several participants described breakdowns in the communication and coordination of transportation services. These systemic failures frequently led to missed appointments, heightened stress, and a general distrust in the reliability of available transportation. Regarding MARTA Mobility, one participant recounted: “*They’ll tell me a time and then they’ll tell the driver, the driver will just get to work or they’ll call the driver in to come get you. They say, “Well, I didn’t know until just now. […] There’s really no communication between the offices, the dispatch, and the drivers*” (P010). Such coordination failures make it difficult for patients to rely on the transportation services they scheduled.

Another participant shared a similar story of a missed appointment when using non-emergency medical transportation provided by their insurance due to what they suspected was a dispatch error:

*I’ve been having problems with them. They’ve been making me miss a couple of appointments. A young lady did show up. I asked her was she there to pick me up. She said, “No, I’m here for Paul.” I’m almost willing to bet she was the one that was supposed to pick me up. It could have been some communication issue between them and the transportation company. I don’t know but I missed my appointment. I’m scheduling on 27th. I have to wait, and I really needed to see my primary care doctor because of the fact that I have this pain in my foot. The foot doctor saw me, so now I know what that pain is. He took X-rays. He said I had arthritis in my foot.* 

(P011)

These accounts underscore how coordination failures between dispatchers, drivers, and patients undermine the effectiveness of transportation programs—despite rides being technically scheduled.

#### 3.1.4. Contextual Differences in Resource Demands

How participants experienced transportation challenges often varied by trip purpose. The two types of trips we focused on, healthcare versus food-related travel, placed different logistical and physical demands on participants, influencing the severity and nature of the transportation barriers they faced.

***Healthcare Trips: Time Sensitivity, Appointment-Based, and Emotional Stress*** 

Healthcare trips are typically arranged by appointment, and missed medical appointments can take a few months to reschedule. This can be critical for patients who have chronic conditions that need timely treatment and adds significant emotional stress to medical-related travel. One participant with heart issues recounted that if she misses her appointment for heart failure, she might get another one in two months. She already missed too many medical appointments because the non-emergency medical transportation provided by insurance did not arrive at the right time (P008).

Patients who own private vehicles also experienced transportation-related difficulties, particularly in navigating traffic and securing affordable parking at healthcare facilities. Traffic congestion on the way to appointments and limited on-site parking were mentioned as recurring stressors. One participant described how hospital parking lots were often full, requiring them to search for alternative parking options that carried high fees. The participant expressed frustration at the lack of affordable options and suggested that hospitals could consider waiving parking fees or offering discounts to patients in financial need, noting that “*A lot of times we really can’t afford it*” (P009). This participant also emphasized the emotional toll that accompanies traffic and parking situations: “*Normally when you go to the doctor, you’re already tired and frustrated. Your pressure is high. All of those interacts with your health, plus sometimes you’re in traffic and bumper to bumper and stuff like that*” (P009).

***Grocery Trips: Flexible Timing but Physical Burden*** 

While food-related trips were generally seen as more flexible than healthcare appointments, they still posed distinct transportation challenges, particularly for participants with mobility limitations or chronic pain. Several participants used walkers or canes, had undergone surgery, or had trouble walking, making travel with grocery bags physically demanding.

Many described relying on rideshare or assistance from family members when carrying heavy or bulky items. One participant noted: “*I’ll catch the bus there but sometimes I might say—I might get a Lyft because I have a bunch of stuff, I catch a Lyft*” (P006). Another participant used Common Courtesy when physical strain or load size made other options infeasible: “*For the grocery store, If I just don’t feel like walking, or if I have more than five bags and I really need to do some heavy shopping*” (P011). Unlike healthcare visits, which were scheduled and time-sensitive, food trips allowed more flexibility. Participants could postpone or skip a trip depending on how they felt physically or emotionally that day (P033).

While the interviews centered around transportation barriers, many participants shared that their transportation barriers were intertwined with other SDOH, such as financial hardship, housing instability, neighborhood crime, and technology barriers. These concurrent challenges suggest that people experiencing transportation challenges are also vulnerable to other SDOH issues.

### 3.2. Strategies to Overcome Transportation Barriers

Participants adopted a range of strategies to cope with persistent transportation barriers. These strategies reflect efforts to navigate both individual and structural constraints, such as limited physical mobility, financial insecurity, and unreliable transportation services. Coping strategies ranged from proactive planning and resource coordination to reluctant transportation usage.

#### 3.2.1. Individual Planning and Multi-Modal Strategies

Participants often developed trip planning routines tailored to their needs, balancing cost, distance, and urgency. Many used different modes of transportation depending on destination and purpose. One participant described: “*I say if it’s less than 10 miles, then I will take the senior transportation. If it’s more like a more serious type of obligation or appointment that I need to make, I will call my insurance*” (P011). Similarly, another participant described how she varied her transportation use depending on where she needed to go: “*I get around for my doctor appointments, I do WellCare transportation, which is Southeastrans because my insurance pays for it, but when I go to my daughter’s, I call MARTA Mobility. The van at my complex, they take us to and from Walmart and Kroger*” (P010). These decisions suggest strategic use of available options, often shaped by cost and purpose of the trip. Another participant shared a workaround strategy to deal with limited hospital parking: “*I drove to Grady because I had an early morning appointment. I was able to get a space. If I’m not able to find a parking space, I’ll go somewhere else and park and catch the MARTA*” (P021). These accounts reflect strategic flexibility in managing transportation, often shaped by availability, affordability, and the nature of the trip.

#### 3.2.2. Seeking Help from Social Networks

Participants with limited access to public and subsidized transportation options due to physical hardship or financial constraints turned to family, friends, and neighbors for assistance. Arranging transportation through their social networks was often more affordable but depended on their availability. One participant recalled how she began depending on her son after she could no longer ride the bus due to physical issues (P002). Another participant mainly asked his brother-in-law for rides with smaller payments: “*I don’t have the money, and for Lyft and Uber might cost you $60 to go from here from Alpharetta and I could pay my brother-in-law $10, $15, take me up there and bring me back*” (P032). Others also described ride arrangements by asking friends or caretakers: “*My caretaker, somebody, either, I have friends now. I have a lot of support, people from the past they are still in my life. I tell I need to go here; I need to go here. They take a week, or somebody help me too like that*” (P004). While social support reduces financial burdens, it also requires flexibility and patience. As participants noted, they have to wait until riders are available, which may put off grocery shopping for a couple of days.

#### 3.2.3. Reducing Travel Needs

Some participants managed transportation barriers by minimizing the number of trips they made. They scheduled multiple appointments on the same day to limit travel or chose to forgo non-urgent trips altogether when their physical condition or financial situation made travel difficult. One participant scheduled all medical visits on the same day to coordinate with her son: “*What I tried to do is set all my doctor’s appointment for the same day so I know Imma be there all day long. It’s like when you were calling me, I’m like, can we do it on the 25th? Because I know I can’t have a way to come back and forth. What I do is I try to tell my son way ahead of time where my appointments are going to be and he’ll say, “Okay, Imma take you*” (P012).

Some participants avoid out-of-home trips as much as they can. As one participant put it, “Sometimes I can’t afford it, sometimes I just don’t go. I say, okay, I can wait till next week or you wait till month to do it” (P032). Another participant, who lacked access to rideshare apps due to not owning a credit card, described the frustration and complexity of arranging transportation: “*Yes, and find somebody that’s going to charge me reasonably because I don’t have what they call an Uber account, or anything. I don’t have a credit card to make an Uber account or anything. If I do, I have somebody else pay for it, and then I pay them for the Uber. If they come get me, they either have a big truck or something, so they gonna try to charge me, especially with the gas thing going on. It’s just very, very frustrating, so I don’t go anywhere*” (P012).

### 3.3. Healthcare and Food Access During COVID-19

The COVID-19 pandemic altered the daily lives of participants by introducing new obstacles to healthcare and food access while intensifying preexisting barriers. We identified three interrelated challenges: direct disruptions in healthcare and food availability, limited effectiveness of online alternatives, and heightened transportation constraints. These experiences highlight how the pandemic exacerbated vulnerabilities in healthcare and food access for low-income individuals with chronic conditions.

#### 3.3.1. Direct Disruptions to Healthcare and Food Availability

Participants emphasized the immediate impact of COVID-19 on the disruption of healthcare availability. Compared to the pre-pandemic experience, when they could see a doctor for quicker care as needed, they faced stricter appointment systems, longer wait times, and sudden cancellations during the pandemic: “*Scheduling is horrible. Sometimes you call, and it’s like three months waiting. Then after you’ve waited for the two months, they call and tell you they ain’t coming in. Or you’re going to have to call and you reschedule*” (P014). Another participant described how her surgery was canceled because of COVID-19: “*I was so mad! Two and a half years because of COVID, twice because of COVID my surgery got canceled. I came here on a Friday for my pre-op. As soon as I walked through the door, they call me, tell me it was canceled for Monday, I was so mad*” (P010).

Food availability was less severely affected than healthcare, though several participants reported shortages at grocery stores and responded by purchasing items in bulk to prepare for future scarcity, “*They didn’t have what I want. Everything was off the shelf. What I did, I bulked it. I bought in bulk*” (P021), or to reduce trips, “*I wore my mask, and not come in contact with people like that. I still went to the grocery store, the stores I want to go to. When I went, I went for the whole month. I just hey imma get this, I ain’t coming back out*” (P006).

#### 3.3.2. Telemedicine and Digital Access Limitations

In response to the restrictions on in-person visits during the pandemic, telemedicine, online shopping, and delivery services saw rapidly increased adoption as alternatives. Nevertheless, low-income patients with chronic conditions often face additional challenges accessing telemedicine and other online services. Several participants recounted telemedicine being unsatisfactory, particularly when physical examination was necessary: “*I’ve done [virtual visits], but dealing with the issues that I’m dealing with, it’s really nothing that can be virtual. You talking to the doctor, he really need to see*” (P014).

Participants also tried grocery deliveries as a safer and more convenient way to obtain food. “*I did shop online. I started that. It was okay because I liked it. At that time, even now when I get stamps or something in the future, I can do shopping online with a food stamp card*” (P017). However, most participants are not familiar with digital technology, and shifting to online shopping created further barriers to food access. As one participant explained, “*I really don’t use them. I don’t know how to. [...] I don’t know how, so I just never bothered with it*” (P012). Another had planned to take a computer skills class offered by the Goodwill Career Center to learn how to complete tasks online, but the class was canceled due to COVID-19 (P035).

#### 3.3.3. COVID-Related Transportation Constraints

Beyond these systemic disruptions, COVID-19 also intensified existing transportation barriers. Some services, such as housing-complex shuttle vans, were suspended, forcing a participant to seek more costly alternatives: “*When COVID hit and they stopped the van, I had to use Common Courtesy […] I was paying extra bills a month because of that […] That was anywhere from $6 to $8 a month. I know it doesn’t sound like a lot, but when you’re strapped for cash, when you’re on a budget, that takes away from your budget*” (P010).

While public transit ridership plummeted during the pandemic, many of our target participants continued to depend on MARTA in their daily lives [35]. By the time of the interviews, MARTA had restored the initially suspended bus lines [47], although service frequency, capacity, and reliability remained lower than pre-pandemic levels due to COVID-related driver shortages that persisted into later years [48,49]. As shown in Table 1, a significant portion of interviewees continued using public transit for medical and food-related trips, although chronic health conditions put them at higher risks associated with COVID exposure and infections.

Many adapted by taking precautionary measures, particularly mask-wearing or going early in the morning to take a bus before it got too crowded. One participant reflected on how riding transit became a new routine: “*In the beginning I was a little scared of it, but now I bring everything I need, my wipes and I try to wear my mask constantly and Lysol spray. You can’t really worry about it. This is the norm now. You got to deal with it or it’s going to drive you crazy*” (P013). Another participant described avoiding public transportation altogether: “*I was depressed during the pandemic; it was boring because I was scared to go places. […] Me and my daughter, she would come and get me, but I was just scared to get on the bus. I don’t think I rode the bus then because that was too close*” (P003). These experiences underscore that while public transportation remained indispensable, the pandemic magnified its financial and emotional burdens.

These accounts show how COVID-19 not only disrupted healthcare and food availability but also exposed the limits of online alternatives and intensified transportation barriers. Overall, the pandemic deepened the existing vulnerabilities faced by low-income individuals with chronic conditions.

## 4. Discussion and Conclusions

Transportation is a critical social determinant of health that interacts with many other SDOHs [10,11]. An important pathway through which transportation challenges affect health is by limiting accessibility to healthcare and healthy food, especially for individuals with diet-related chronic health conditions. This study examines the transportation barriers facing low-income individuals with diabetes and hypertension in accessing healthcare, participating in FAM, and obtaining healthy food as part of their disease management plan. Using in-depth interviews and thematic analysis, we identified three recurrent themes regarding barriers that participants described as limiting their healthcare and food access: high transportation costs, long wait times and delays, and unreliable services due to coordination issues or systemic inefficiencies. These barriers, acting independently or in combination, restricted participants’ timely access to healthcare and food resources and often led to frustration and missed medical appointments. Such experiences diminished trust in the reliability of transportation services and ultimately disrupted treatment adherence and timely care, threatening effective chronic disease management. As described earlier in the paper, unreliable transportation leads to missed appointments, with rescheduled appointments often delayed for months and sometimes incurring late fees. These disruptions compromise continuity and adherence to care and add further burdens that may diminish patients’ confidence in the healthcare system’s ability to coordinate timely care. In addition, delays or failures in non-emergency medical transportation provided by insurance directly weaken trust in using these services, as patients may perceive these lapses as failures within a service designed to facilitate healthcare access. As illustrated in Figure 1, transportation barriers directly affect healthcare access and utilization by disrupting continuity and adherence to care, ultimately influencing health outcomes.

In healthcare settings, care delays are associated with increased hospitalization burden [18,19,50], underscoring the time-sensitive nature of healthcare utilization. Given that transportation plays a critical role in timely care [4,5,17], transportation should be recognized as a key social determinant of health requiring upstream intervention from a public health perspective.

We also examined the coping strategies participants use to overcome transportation barriers, and how the COVID-19 pandemic reshaped or compounded their transportation challenges. Participants also described how they experienced and managed transportation barriers differently between medical visits and grocery trips—the two primary types of out-of-home activities in this study. Medical visits often involve time-sensitive schedules with emotional stress, whereas grocery trips can be flexible but require handling bulky items.

Participants’ accounts indicated that the COVID-19 pandemic further disrupted their healthcare and food access during the study period. Some participants avoided public transportation due to fear of infection, while others continued riding transit with precautionary measures. While healthcare and other services shifted online in response to the pandemic and subsequent public health restrictions, adoption of those virtual alternatives remained low among our target population due to dissatisfaction, distrust, and limited digital literacy that constrained their ability to access online services and resources. This finding underscores that the pandemic exacerbated the vulnerabilities of those dependent on public transportation and unfamiliar with online activities.

Several limitations should be acknowledged. First, this study’s 36 interview participants from the FAM program limit the generalizability of our findings to all FAM participants. Our recruitment strategies may have introduced selection bias, potentially overrepresenting participants who were more easily reachable by phone or more actively engaged in the FAM program. Although the food pharmacy patient navigator assisted with on-site recruitment, interview scheduling relied on phone communication, which may have limited participation among individuals who could not be reached by phone. Comparable demographic data for the overall FAM population were not publicly available, which constrained our ability to assess how closely the interview sample reflects the broader FAM population.

Second, because participants were recruited through FAM, the findings primarily reflect the experiences of patients already engaged in care and nutrition support and may have limited transferability beyond this context. Future research should employ complementary strategies—such as partnering with community organizations, engaging non-clinical settings, and offering flexible participation modalities—to enhance inclusion of more marginalized populations.

Third, interviewer characteristics may have influenced participants’ responses or willingness to share their experiences [51]. Although such effects cannot be fully controlled, we sought to reduce potential bias through collaborative coding and team discussions that emphasized consistency and collective agreement in code application, ensuring balanced interpretation of the data.

Nevertheless, the recurrent patterns identified in this study are consistent with previous research on transportation barriers in healthcare and food access [2,4,7], supporting the credibility and transferability of the research findings to similar contexts. Moreover, the depth and richness of the qualitative data provide valuable insights into the lived experiences and coping mechanisms of individuals navigating these barriers.

This study contributes to the literature on healthcare access and transportation as an SDOH by discussing the transportation barriers to healthcare and food access, as well as examining how individuals manage or respond to these challenges. Participants in this study employed a range of strategies, from carefully planning trips with consideration of trip purpose and timing to seeking help from family or friends. Meanwhile, some avoided going out and chose to stay at home as much as possible, often at the expense of their healthcare and disease management.

Based on participants’ experiences highlighting the importance of transportation access for managing chronic conditions, we suggest the following recommendations. First, a wider range of transportation options is needed to accommodate diverse health, mobility, and financial circumstances. People with chronic illnesses face different physical and economic constraints, making it difficult to rely on public transportation or private vehicles as universal solutions. For example, healthcare institutions can ease access by offering subsidies for gas and parking fees [17]. Transit agencies implementing fare caps could improve equitable transportation access for low-income individuals [52]. For instance, Portland’s TriMet system waives fares after reaching daily ($5.60) and monthly ($100) spending caps, with reduced rates for seniors, Medicare recipients, and people with disabilities [53]. In addition, transit agencies should provide reliable paratransit or on-demand shuttle services [7].

Non-emergency medical transportation provided by insurance should expand to include other essential trips [54], such as visits to FAM sites and grocery stores with fresh produce. Local governments can broaden rideshare-based transportation programs for seniors and low-income individuals and increase trip limits within these programs. Expanding these programs will allow individuals to select the mode that best fits their needs, ensuring that transportation assistance is flexible and responsive [55]. Ultimately, these efforts can help reduce missed or delayed appointments and improve treatment adherence, which are critical for effective chronic disease management.

Second, transportation agencies and healthcare institutions need to strengthen their collaboration and coordination to ensure seamless access to care [17,54]. This may involve a more streamlined process for eligible patients to apply for and receive transportation assistance and subsidies [54], better accessibility support at transit stops near healthcare facilities, coordinated scheduling for medical appointments and driver pickups [2], and technology assistance for utilizing emerging services [2], such as telemedicine, ride-hailing, and delivery apps.

Specifically, healthcare institutions are encouraged to train and deploy digital navigators who can help patients effectively use smartphones and online resources for health management [56,57]. While digital solutions such as telemedicine and online grocery ordering became increasingly common during and after the pandemic, many participants faced challenges using these services due to limited digital literacy. To promote digital equity and support healthy behaviors, healthcare institutions, local governments, and community partners could provide hands-on digital literacy training [58], offer subsidized devices [59], and provide low-cost internet service plans to enhance connectivity and engagement with online resources [58,60]. Meanwhile, maintaining flexible care delivery options that provide both digital and in-person access remains essential for individuals who cannot reliably use digital tools or who are dissatisfied with telemedicine alternatives [60]. Additionally, healthcare institutions should systematically assess individual patients’ digital access and literacy levels [58], and monitor and evaluate the effectiveness and user satisfaction of these digital literacy initiatives across different age, income, and health condition groups to ensure equitable implementation of digital health interventions [61].

In summary, this study highlights the critical role of transportation for healthcare and healthy food access in supporting effective chronic disease management. By examining the transportation barriers individuals encounter and the coping strategies they adopt, our study provides a more comprehensive understanding of how transportation barriers and interventions can shape health management. Finally, this study offers policy recommendations for improving transportation access to support chronic disease management beyond clinical settings.

## Figures and Tables

**Table 1:** Characteristics of Interview Participants (*n* = 36).

Category	*n* (%)
**Employment**	
Employed	7 (19.4%)
Unemployed	22 (61.1%)
Not mentioned	7 (19.4%)
**Gender**	
Female	29 (80.6%)
Male	7 (19.4%)
**Modes of Transportation for Healthcare**	
Public transportation (MARTA bus, train, paratransit)	16 (44.4%)
Drive	11 (30.6%)
Getting a ride	5 (13.9%)
Non-emergency medical transportation provided by insurance	8 (22.2%)
County senior transportation	0 (0.0%)
Walking	1 (2.8%)
Not mentioned	1 (2.8%)
**Modes of Transportation for Food**	
Public transportation (MARTA bus, train, paratransit)	10 (27.8%)
Drive	4 (11.1%)
Getting a ride	8 (22.2%)
Non-emergency medical transportation provided by insurance	1 (2.8%)
County senior transportation	2 (5.6%)
Complex Van	1 (2.8%)
Walking	4 (11.1%)
Online delivery	4 (11.1%)
Not mentioned	9 (25%)

Note. For modes of transportation, participants could report multiple modes (e.g., driving to a MARTA station and then taking MARTA); therefore, totals exceed 36 (100%).

## Data Availability

The interview transcripts are not publicly available due to privacy and ethical restrictions.

## Supplementary Materials

The following supporting information can be downloaded at: https://www.mdpi.com/article/10.3390/healthcare13222869/s1, Table S1. Code Frequency Summary for Selected Key Codes.; Table S2. Code Co-occurrence Matrix for Selected Key Codes.; Figure S1. Tree map of Selected Key Codes.; Table S3: Checklist based on the Consolidated Criteria for Reporting Qualitative Research.
