# Supplementary material for: Overcoming Transportation Barriers for Low-Income Individuals with Chronic Conditions: Identifying Barriers and Strategies in Access to Healthcare and Food as Medicine (FAM)
Source: Healthcare (Basel). 2025 Nov 11;13(22):2869. doi: 10.3390/healthcare13222869 (PMC12652909; doi:10.3390/healthcare13222869)
Supplement: Supplementary file 1 [file healthcare-13-02869-s001.zip › healthcare-3924180-supplementary.pdf]

Table S1. Code Frequency Summary for Selected Key Codes.

Categories	Hierarchical Name	Name	Description	# Coded Files	# Coded References	# Coded Words	# Coded Paragraphs
1. Transportation Barriers	Daily Life\Getting around\Barriers and Strategies\Cost of transportation	Cost of transportation	Fare, gas, parking, car costs, etc.	18	44	18,230	686
1. Transportation Barriers	Daily Life\Getting around\Barriers and Strategies\Distance	Distance		15	36	9,739	489
1. Transportation Barriers	Daily Life\Getting around\Barriers and Strategies\Time	Time	Timing, time costs, delays	21	40	16,675	742
1. Transportation Barriers	Health\Health issues\Disability or mobility constraints	Disability or mobility constraints	Can't walk, can't drive, walking aids, wheelchair, etc.	27	109	38,835	1450
1. Transportation Barriers	Life Circumstances\Money issues	Money issues	Costs, expenses, can't afford things, income, etc.	30	165	44,304	1976
1. Transportation Barriers	Life Circumstances\Safety	Safety		17	62	21,932	792
1. Transportation Barriers	Systems\Cross-System Issues	Cross-System Issues		22	98	47,158	1649
1. Transportation Barriers	Systems\Medical\Appointments\Coming to appointments	Coming to appointments	Transportation, parking, traffic, etc.	30	77	24,619	1090
2. Coping Strategies	Daily Life\Getting around\Barriers and Strategies\Modal choice	Modal choice	deciding which travel mode(s) to use: cost, distance, carrying stuff, weather, availability of help, etc.	28	75	24,874	1055
2. Coping Strategies	Daily Life\Getting around\Barriers and Strategies\Not going to places	Not going to places	Canceling plans or choosing closer locations due to lack of transportation, staying home	16	37	8,608	353
2. Coping Strategies	Daily Life\Getting around\Barriers and Strategies\Trip planning	Trip planning	Planning ahead before going to places, strategies, trip chaining, combining activities	23	76	34,745	1472
2. Coping Strategies	Daily Life\Getting around\Getting a ride	Getting a ride	From family, friends, or paid	26	95	25,555	1020

			driver (including taxi and rideshare)				
2. Coping Strategies	Social Relationships\Social support\Seeking or getting help	Seeking or getting help		24	86	27,058	1009
3. Pandemic Impacts	COVID\Behavioral Changes	Behavioral Changes	Changes in lifestyle, travel modes, social interactions, etc.	31	112	29,227	1240
3. Pandemic Impacts	COVID\Pandemic Impacts\Medical care	Medical care	Pandemic's impact on getting medical care, scheduling, wait time, canceled surgery, etc.	25	54	14,216	530
3. Pandemic Impacts	COVID\Prevention Strategies\COVID requirements	COVID requirements	Requirements by systems/agencies/venues the respondent uses, e.g., mask requirements, screening, temperature checks	9	21	3,519	186
3. Pandemic Impacts	COVID\Prevention Strategies\Self prevention	Self-prevention	What the respondent does to prevent COVID, e.g., not going out, wearing a mask, washing hands	31	82	21,971	940
4. Technology barriers and solutions	Technologies\Doing things online\Telehealth	Telehealth	Virtual doctor visits, seeking health advice online, using patient portals or messaging health providers	12	26	7,301	339
4. Technology barriers and solutions	Technologies\Technology barriers	Technology barriers	(other than access and cost)	19	52	14,117	652
5. Modes and Services	Systems\Food\FAM	FAM	Food as Medicine	32	140	51,745	2276
5. Modes and Services	Systems\Food\Grocery shopping\Grocery stores	Grocery stores		28	54	19,484	905
5. Modes and Services	Systems\Medical	Medical	Healthcare system	35	600	252,766	10375
5. Modes and Services	Systems\Transportation\Other transportation programs	Other transportation programs		19	99	29,053	1206

5. Modes and Services	Systems\Transportation\Other transportation programs\Common Courtesy	Common Courtesy	Fulton County's senior transportation program, dollar ride	6	24	5,162	236
5. Modes and Services	Systems\Transportation\Other transportation programs\Grady non-emergency transport	Grady non-emergency transport		5	7	3,445	166
5. Modes and Services	Systems\Transportation\Other transportation programs\Non-emergency medical transportation provided by insurance	Non-emergency medical transportation provided by insurance	Southeastrans, WellCare, Humana, etc.	11	36	12,349	461
5. Modes and Services	Systems\Transportation\Public transit	Public transit	MARTA or other public transit systems	32	336	105,400	4406
5. Modes and Services	Systems\Transportation\Public transit\MARTA Mobility	MARTA Mobility		15	97	30,119	1265
5. Modes and Services	Systems\Transportation\Public transit\Reduced Fare	Reduced Fare		11	28	7,540	341
5. Modes and Services	Systems\Transportation\Rideshare	Rideshare		24	55	19,522	803

Note. For brevity, this table presents a subset of key codes. The complete code frequency summary can be provided upon request.

Table S2. Code Co-occurrence Matrix for Selected Key Codes.

Categories	Code co-occurring frequency by reference (numbers in parentheses are frequency by interviewee)	Cost of transportation	Distance	Time	Disability or mobility constraints
1. Transportation Barriers	Daily Life\Getting around\Barriers and Strategies\Cost of transportation	38 (18)	8 (5)	9 (5)	5 (5)
1. Transportation Barriers	Daily Life\Getting around\Barriers and Strategies\Distance	8 (5)	30 (15)	15 (11)	5 (5)
1. Transportation Barriers	Daily Life\Getting around\Barriers and Strategies\Time	9 (5)	15 (11)	34 (21)	9 (8)
1. Transportation Barriers	Health\Health issues\Disability or mobility constraints	5 (5)	5 (5)	9 (8)	77 (27)
1. Transportation Barriers	Life Circumstances\Money issues	20 (9)	7 (4)	8 (5)	9 (7)
1. Transportation Barriers	Life Circumstances\Safety	1 (1)	3 (3)	5 (5)	3 (3)
1. Transportation Barriers	Systems\Cross-System Issues	4 (4)	2 (2)	11 (10)	10 (8)
1. Transportation Barriers	Systems\Medical\Appointments\Coming to appointments	11 (8)	7 (6)	8 (7)	12 (10)
2. Coping Strategies	Daily Life\Getting around\Barriers and Strategies\Modal choice	12 (9)	17 (13)	24 (19)	21 (13)
2. Coping Strategies	Daily Life\Getting around\Barriers and Strategies\Not going to places	6 (5)	3 (2)	2 (2)	4 (4)
2. Coping Strategies	Daily Life\Getting around\Barriers and Strategies\Trip planning	21 (12)	16 (11)	21 (14)	17 (10)
2. Coping Strategies	Daily Life\Getting around\Getting a ride	16 (10)	5 (5)	10 (9)	18 (12)
2. Coping Strategies	Social Relationships\Social support\Seeking or getting help	7 (5)	0 (0)	0 (0)	15 (8)
3. Pandemic Impacts	COVID\Behavioral Changes	1 (1)	0 (0)	1 (1)	3 (3)
3. Pandemic Impacts	COVID\Pandemic Impacts\Medical care	0 (0)	0 (0)	1 (1)	3 (3)
3. Pandemic Impacts	COVID\Prevention Strategies\COVID requirements	1 (1)	0 (0)	0 (0)	0 (0)
3. Pandemic Impacts	COVID\Prevention Strategies\Self prevention	2 (2)	1 (1)	1 (1)	2 (2)
4. Technology barriers and solutions	Technologies\Doing things online\Telehealth	0 (0)	0 (0)	0 (0)	2 (2)
4. Technology barriers and solutions	Technologies\Technology barriers	1 (1)	0 (0)	1 (1)	3 (2)
5. Modes and Services	Systems\Food\FAM	8 (7)	5 (4)	6 (6)	10 (9)
5. Modes and Services	Systems\Food\Grocery shopping\Grocery stores	6 (4)	2 (2)	5 (5)	15 (12)
5. Modes and Services	Systems\Medical	18 (12)	8 (6)	13 (12)	32 (20)
5. Modes and Services	Systems\Transportation\Other transportation programs	9 (5)	5 (5)	5 (5)	12 (9)

5. Modes and Services	Systems\Transportation\Other transportation programs\Common Courtesy	4 (3)	1 (1)	0 (0)	0 (0)
5. Modes and Services	Systems\Transportation\Other transportation programs\Grady non-emergency transport	3 (3)	0 (0)	0 (0)	2 (2)
5. Modes and Services	Systems\Transportation\Other transportation programs\Non-emergency medical transportation provided by insurance	4 (3)	4 (4)	4 (4)	9 (7)
5. Modes and Services	Systems\Transportation\Public transit	16 (12)	15 (10)	21 (15)	35 (17)
5. Modes and Services	Systems\Transportation\Public transit\MARTA Mobility	5 (5)	3 (3)	9 (5)	21 (11)
5. Modes and Services	Systems\Transportation\Public transit\Reduced Fare	6 (5)	3 (3)	1 (1)	5 (4)
5. Modes and Services	Systems\Transportation\Rideshare	9 (7)	5 (5)	10 (8)	9 (8)

Note. For brevity, a partial set of key codes is presented. The complete co-occurrence matrix can be provided upon request.

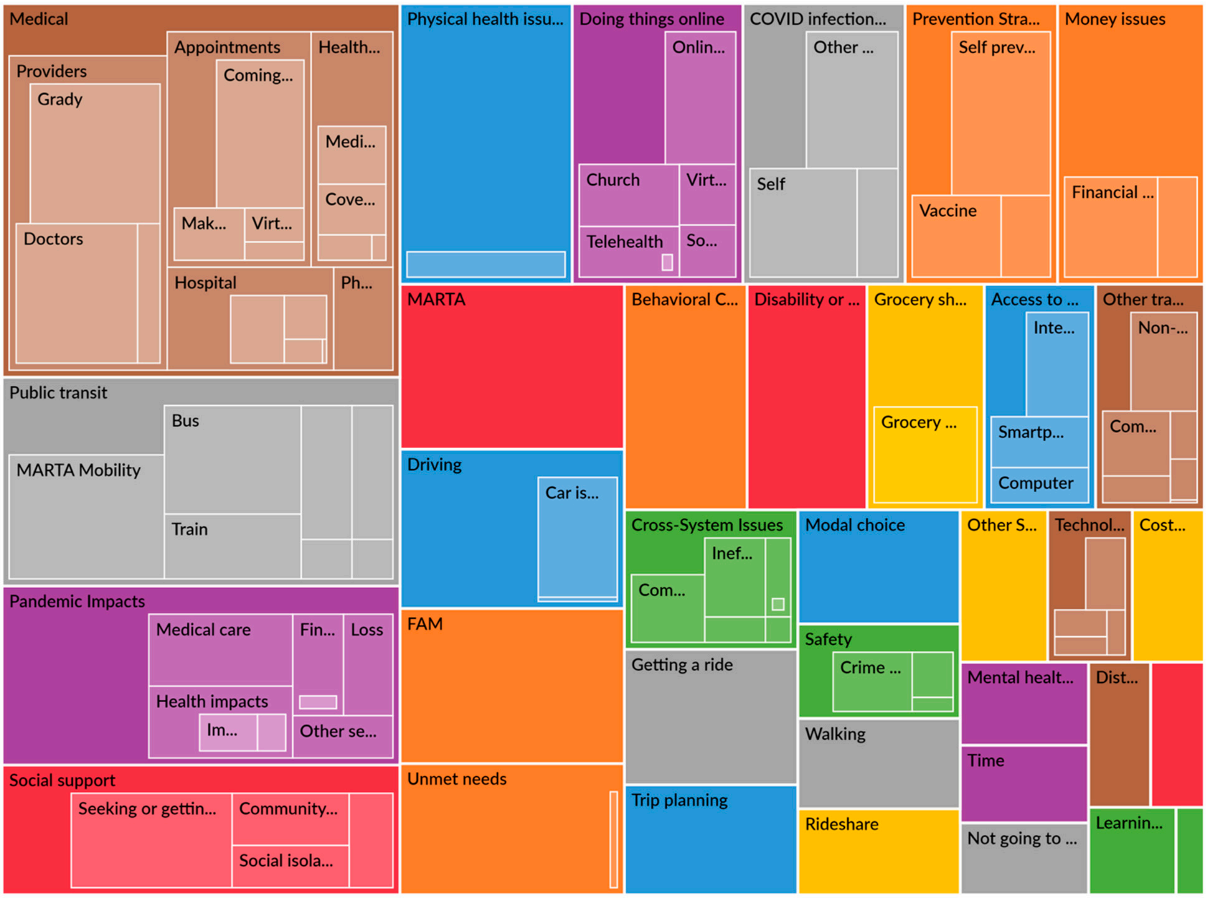

Figure S1. Tree map of Selected Key Codes.

Table S3: Checklist based on the Consolidated Criteria for Reporting Qualitative Research.

No Item	Guide questions/description	Response	Location in Manuscript
Domain 1: Research team and reflexivity			
Personal Characteristics			
1. Interviewer/facilitator	Which author/s conducted the interview or focus group?	SS and JL conducted all interviews.	—
2. Credentials	What were the researcher's credentials?	SS and JL were doctoral students in the Department of Sociology at Georgia State University.	—
3. Occupation	What was their occupation at the time of the study?	Both SS and JL were doctoral students and served as graduate research assistants on the project.	—
4. Gender	Was the researcher male or female?	Both interviewers (SS and JL) were female.	—
5. Experience and training	What experience or training did the researcher have?	Interviewers were graduate students trained in qualitative research methods.	—
Relationship with participants			
6. Relationship established	Was a relationship established prior to study commencement?	Because this qualitative study was linked to a broader transportation research project (see Section 2.2 Data Collection), a few participants had prior contact with SS, who had conducted counseling sessions as part of that transportation research project. These interactions were limited to research-related counseling and did not involve clinical care or ongoing personal relationships.	—
7. Participant knowledge of the interviewer	What did the participants know about the researcher?	Participants were informed that the interviewers were part of a university research team studying transportation and healthcare access and were independent from participants' healthcare providers. The study's	—

		purpose was explained during the informed consent process.	
8. Interviewer Characteristics	What characteristics were reported about the interviewer/facilitator?	The interviewers were trained graduate researchers with an academic interest in transportation and healthcare access. They had no personal relationship with participants.	—
Domain 2: study design			
Theoretical framework			
9. Methodological orientation and Theory	What methodological orientation was stated to underpin the study?	Thematic analysis with an inductive, data-driven approach.	Section 2.3 Data Analysis
Participant selection			
10. Sampling	How were participants selected?	Participants were selected through purposive sampling.	Section 2.2 Data Collection
11. Method of approach	How were participants approached?	Participants were approached via telephone outreach and flyers distributed at the Food as Medicine site.	Section 2.2 Data Collection
12. Sample size	How many participants were in the study?	Thirty-six participants were interviewed.	Section 2.2 Data Collection
13. Non-participation	How many people refused to participate or dropped out? Reasons?	Numbers of refusals and reasons were not recorded; interviews were conducted with those who could be contacted and agreed to participate.	—
Setting			
14. Setting of data collection	Where was the data collected?	Interviews were conducted either in a private room at the Grady Food Pharmacy or in a university office.	—
15. Presence of non-participants	Was anyone else present besides the participants and researchers?	Occasionally, a family member was present at the participant's request to provide mobility assistance.	—
16. Description of sample	What are the important characteristics of the sample?	Limited demographic information, including gender and employment status, was collected; other characteristics were not recorded.	Table 2
Data collection			

17. Interview guide	Were questions, prompts, guides provided by the authors? Was it pilot tested?	Semi-structured interviews focused on transportation use, experiences with the FAM program, and the impacts of the COVID-19 pandemic. The guide was not pilot tested but was practiced within the research team.	Section 2.2 Data Collection
18. Repeat interviews	Were repeat interviews carried out? If yes, how many?	No.	—
19. Audio/visual recording	Did the research use audio or visual recording to collect the data?	Interviews were audio-recorded.	Section 2.3 Data Analysis
20. Field notes	Were field notes made during and/or after the interview or focus group?	No field notes were made.	—
21. Duration	What was the duration of the interviews or focus group?	Each interview lasted approximately one hour.	Section 2.2 Data Collection
22. Data saturation	Was data saturation discussed?	Thematic saturation was reached when no new codes emerged and was confirmed through team discussion.	Section 2.3 Data Analysis
23. Transcripts returned	Were transcripts returned to participants for comment and/or correction?	No transcripts were returned to participants.	—
Domain 3: analysis and findings			
Data analysis			
24. Number of data coders	How many data coders coded the data?	Six researchers coded the data.	See Section 2.3 Data Analysis
25. Description of the coding tree	Did authors provide a description of the coding tree?	The supplementary materials (Table S1 and Figure S1) describe the coding tree for the selected key codes, showing their hierarchical names and a tree map visualization.	—

26. Derivation of themes	Were themes identified in advance or derived from the data?	Themes were derived from participants' responses.	Section 2.3 Data Analysis
27. Software	What software, if applicable, was used to manage the data?	NVivo 14.24.3	Section 2.3 Data Analysis
28. Participant checking	Did participants provide feedback on the findings?	No participant checking was conducted.	—
Reporting			
29. Quotations presented	Were participant quotations presented to illustrate the themes / findings? Was each quotation identified?	Participant quotations are presented and identified by assigned participant codes (e.g., P010).	Section 3. Results
30. Data and findings consistent	Was there consistency between the data presented and the findings?	Yes, the data presented are consistent with the reported findings.	Section 3. Results
31. Clarity of major themes	Were major themes clearly presented in the findings?	Yes, major themes are clearly presented.	Section 3. Results
32. Clarity of minor themes	Is there a description of diverse cases or discussion of minor themes?	Quotations reflect variation within major themes, although minor themes were not analyzed separately.	—

Adapted from: Tong, A.; Sainsbury, P.; Craig, J. Consolidated Criteria for Reporting Qualitative Research (COREQ): A 32-Item Checklist for Interviews and Focus Groups. *Int. J. Qual. Health Care*, **2007**, *19* (6), 349–357.
